# IκBζ facilitates protective immunity against *Salmonella* infection via Th1 differentiation and IgG production

**DOI:** 10.1038/s41598-019-44019-3

**Published:** 2019-06-10

**Authors:** Jae-Hee Ahn, Jaewon Cho, Bo-Eun Kwon, Geun-Shik Lee, Sung-il Yoon, Seung Goo Kang, Pyeung-Hyeun Kim, Mi-Na Kweon, Hyungjun Yang, Bruce A. Vallance, Young-In Kim, Sun-Young Chang, Hyun-Jeong Ko

**Affiliations:** 1 Laboratory of Microbiology and Immunology, College of Pharmacy, Kangwon National University, Chuncheon, 24341 Republic of Korea; 2 College of Veterinary Medicine, Kangwon National University, Chuncheon, 24341 Republic of Korea; 3 Division of Biomedical Convergence, School of Biomedical Science and Institute of Bioscience and Biotechnology, Kangwon National University, Chuncheon, 24341 Republic of Korea; 4 Department of Molecular Bioscience, School of Biomedical Science and Institute of Bioscience and Biotechnology, Kangwon National University, Chuncheon, 24341 Republic of Korea; 5 Mucosal Immunology Laboratory, Department of Convergence Medicine, University of Ulsan College of Medicine/Asan Medical Center, Seoul, Republic of Korea; 6 Department of Pediatrics, British Columbia Children’s Hospital, University of British Columbia, Vancouver, British Columbia Canada; 7 Laboratory of Microbiology, College of Pharmacy, Ajou University, Suwon, 16499 Republic of Korea

**Keywords:** Antimicrobial responses, Bacterial infection, Live attenuated vaccines

## Abstract

Inhibitor of kappa B (IκB)-ζ transcription is rapidly induced by stimulation with TLR ligands and IL-1. Despite high IκBζ expression in inflammation sites, the association of IκBζ with host defence via systemic immune responses against bacterial infection remains unclear. Oral immunisation with a recombinant attenuated *Salmonella* vaccine (RASV) strain did not protect IκBζ-deficient mice against a lethal *Salmonella* challenge. IκBζ-deficient mice failed to produce *Salmonella* LPS-specific IgG, especially IgG2a, although inflammatory cytokine production and immune cell infiltration into the liver increased after oral RASV administration. Moreover, IκBζ-deficient mice exhibited enhanced splenic germinal centre reactions followed by increased total IgG production, despite IκBζ-deficient B cells having an intrinsic antibody class switching defect. IκBζ-deficient CD4^+^ T cells poorly differentiated into Th1 cells. IFN-γ production by CD4^+^ T cells from IκBζ-deficient mice immunised with RASV significantly decreased after restimulation with heat-killed RASV *in vitro*, suggesting that IκBζ-deficient mice failed to mount protective immune responses against *Salmonella* infection because of insufficient Th1 and IgG production. Therefore, IκBζ is crucial in protecting against *Salmonella* infection by inducing Th1 differentiation followed by IgG production.

## Introduction

Inhibitor of kappa B (IκB)-ζ is a protein encoded by the NF-kappa-B inhibitor zeta (*NFKBIZ*) gene, and it contains ankyrin repeat domains and is a member of the IκB family of nuclear proteins^1–3^. Activation of IκBζ in macrophages leads to IL-6 production, which is known to be mediated by the Myd88 (myeloid differentiation primary response 88) adaptor molecule^4^. The depletion of IκBζ reduces the production of Myd88-dependent IL-6 production in various cell types, including macrophages, mouse embryonic fibroblasts, and epithelial cells. The transcription of IκBζ is rapidly induced by TLR (Toll-like receptor) stimulation and IL-1 signalling^2,5^. Interestingly, it was reported that there were multiple *NFKBIZ* polymorphisms associated with susceptibility to *Streptococcus pneumonia*-mediated invasive pneumococcal disease^6^. Moreover, *Legionella pneumophilia* infection was found to induce IκBζ-dependent IL-6 expression in lung epithelial cells^7^.

It is well known that IκBζ is highly expressed in inflammation sites^8^; however, the association between the IκBζ molecule and systemic immune response has not been thoroughly investigated. Several reports have suggested a cell-specific role of IκBζ. The lack of IκBζ in B cells reduces the antibody response, especially TLR-mediated T cell-independent class switch recombination (CSR) in B cells^9^. In addition, it was reported that IκBζ was required for the development of Th17 cells and that IκBζ-deficient mice were resistant to experimental autoimmune encephalomyelitis because of a CD4^+^ T-cell intrinsic defect in Th17 development^3^. IκBζ plays a crucial role in natural killer cell activation in response to IL-12 and IL-18^10^. More importantly, IκBζ is an essential activator of IL-10 expression in macrophages after LPS stimulation; thus, it regulates expression of anti-inflammatory cytokine and pro-inflammtory cytokines, including IL-6, IL-12, and CCL2^11,12^. Despite the reduced IL-6 production, the release of TNF-α is not affected or increased by other TLR ligands, including zymosan and peptidoglycan in IκBζ-deficient macrophages^11,12^.

Thus, we presume that the uncontrolled secretion of pro-inflammatory cytokines might be associated with the development of chronic inflammation found in IκBζ-deficient mice.

In our previous study, we showed that oral administration of a recombinant attenuated *Salmonella* vaccine strain (RASV) in mice elicited sufficient immune responses, including LPS-specific Ab responses, to protect virulent *Salmonella* infection in wild-type mice^13–15^. However, RASV vaccination did not protect IκBζ^−/−^ mice against virulent *Salmonella* infection. Therefore, we further assessed which components of the immune responses associated with IκBζ are critical to establishing protective immunity against *Salmonella* infection after oral RASV immunisation.

## Results

### Attenuated *Salmonella* vaccines did not protect IκBζ^−/−^ mice from virulent *Salmonella* infection

**Figure 1:**
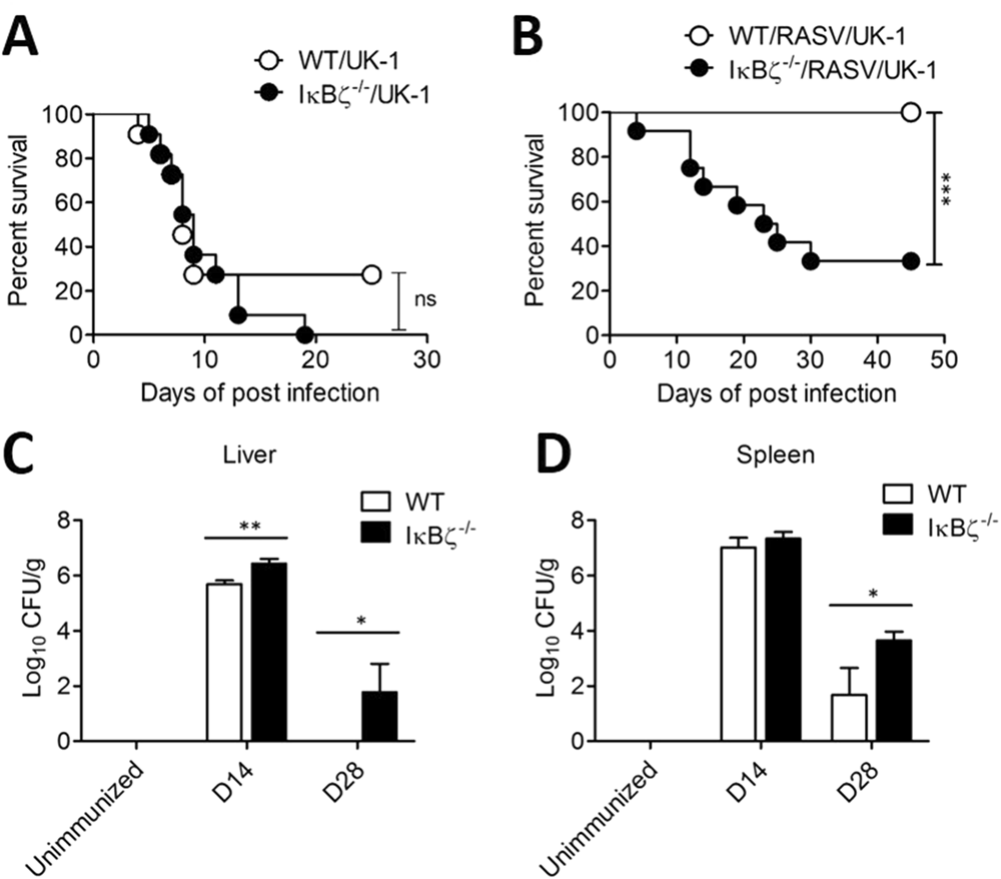
IκBζ^−/−^ mice exhibited enhanced susceptibility after challenge with lethal *Salmonella* infection even following pre-vaccination. (**A**) Wild-type (WT) and IκBζ^−/−^ mice were orally challenged with 10^7^ CFU of a lethal wild-type *Salmonella* strain (UK-1) per mouse (n = 11/group). ns; not significant (log-rank test). Experiment came to the end at 25 days of post infection. Survived mice was sacrificed. (**B**) Wild-type and IκBζ^−/−^ mice were immunised by oral administration with 10^9^ CFU of a recombinant attenuated *Salmonella* vaccine strain (RASV) per mouse twice at 2-week intervals. Mice were challenged with UK-1 at 10^7^ CFU per mouse 14 d after the final RASV oral immunisation. The survival of mice was monitored following challenge (n = 13 for WT/RASV/UK-1, n = 12 for IκBζ^−/−^/RASV/UK-1; ^***^P < 0.001, based on log-rank test). (**C**,**D**) Wild-type and IκBζ^−/−^ mice were immunised with oral administration of 10^9^ CFU of RASV per mouse twice at 2-week intervals. (**C**) Liver tissues and (**D**) spleens from the mice were homogenised to determine the CFU of RASV at unimmunised, 14 d (D14) after the first administration with RASV, and 14 d after the second administration (D28). Data are representative of three independent experiments. ns, not significant; ^*^P < 0.05, ^**^P < 0.01, and ^***^P < 0.001 based on ANOVA with Bonferroni’s multiple comparison test.

To evaluate the susceptibility of IκBζ-deficient (IκBζ^−/−^) mice against *Salmonella* infection, we administered virulent *Salmonella* *typhimurium* (UK-1) to wild-type and IκBζ^−/−^ mice. In non-vaccinated mice, the survival rate of IκBζ^−/−^ mice was not significantly different from that of WT (*p = 0*.*4550*, log-rank test), suggesting that innate immunity is critical for the early survival of non-vaccinated mice after *Salmonella* infection regardless of the existence of IκBζ (Fig. 1A). Further, we conducted assay determining *Salmonella* CFU from liver and spleen of the UK-1-infected mice. At day 9 after oral administration of 10^7^ CFU per mice, all mice administered with UK-1 showed *Salmonella* colony formation in liver and spleen of IκBζ^−/−^ mice and WT mice (Supplementary Fig. 1).

Next, to determine whether adaptive immunity could render protection against virulent *Salmonella* challenge in IκBζ^−/−^ mice, we adopted a vaccine model using an attenuated *Salmonella* vaccine strain, RASV, in accordance with a previous study^14^. RASV was orally administered to wild-type or IκBζ^−/−^ mice at a dose of 10^9^ CFU/mouse. After 14 d, each mouse was secondly immunised with the same dose of RASV. At 14 d after the second immunisation, mice were orally challenged with 10^7^ CFU of virulent *Salmonella* UK-1. Although oral vaccination with RASV successfully protected wild-type mice against virulent *Salmonella* infection, only 40% of IκBζ^−/−^ mice survived (Fig. 1B) and the survival of RASV-vaccinated mice was significantly different (p = 0.0003, log-rank test). This result suggested that IκBζ^−/−^ mice failed to mount sufficient protective immunity against *Salmonella* infection.

Despite the high attenuation of the vaccine strain, immune-compromised hosts contain defects in the clearance of live vaccine strains and sustain continued colonisation^16^. Likewise, the residual attenuated RASV was identified in the spleen and liver at unimmunised and 14 d (D14) after the first administration with RASV and 14 d after the second administration (D28). There was more colonisation of *Salmonella* in the liver at 14 d after the first RASV administration in IκBζ^−/−^ mice than in wild-type mice (Fig. 1C). Furthermore, at 14 d (D28) after the second RASV administration, *Salmonella* was still detected in liver and spleen tissues in IκBζ-deficient mice (Fig. 1C,D). These results suggested that IκBζ^−/−^ mice could not efficiently eliminate attenuated *Salmonella* after oral administration, possibly resulting in the insufficient protective efficacy of RASV in IκBζ^−/−^ mice.

### IκBζ^−/−^ led to chronic inflammation after oral administration of the RASV strain

**Figure 2:**
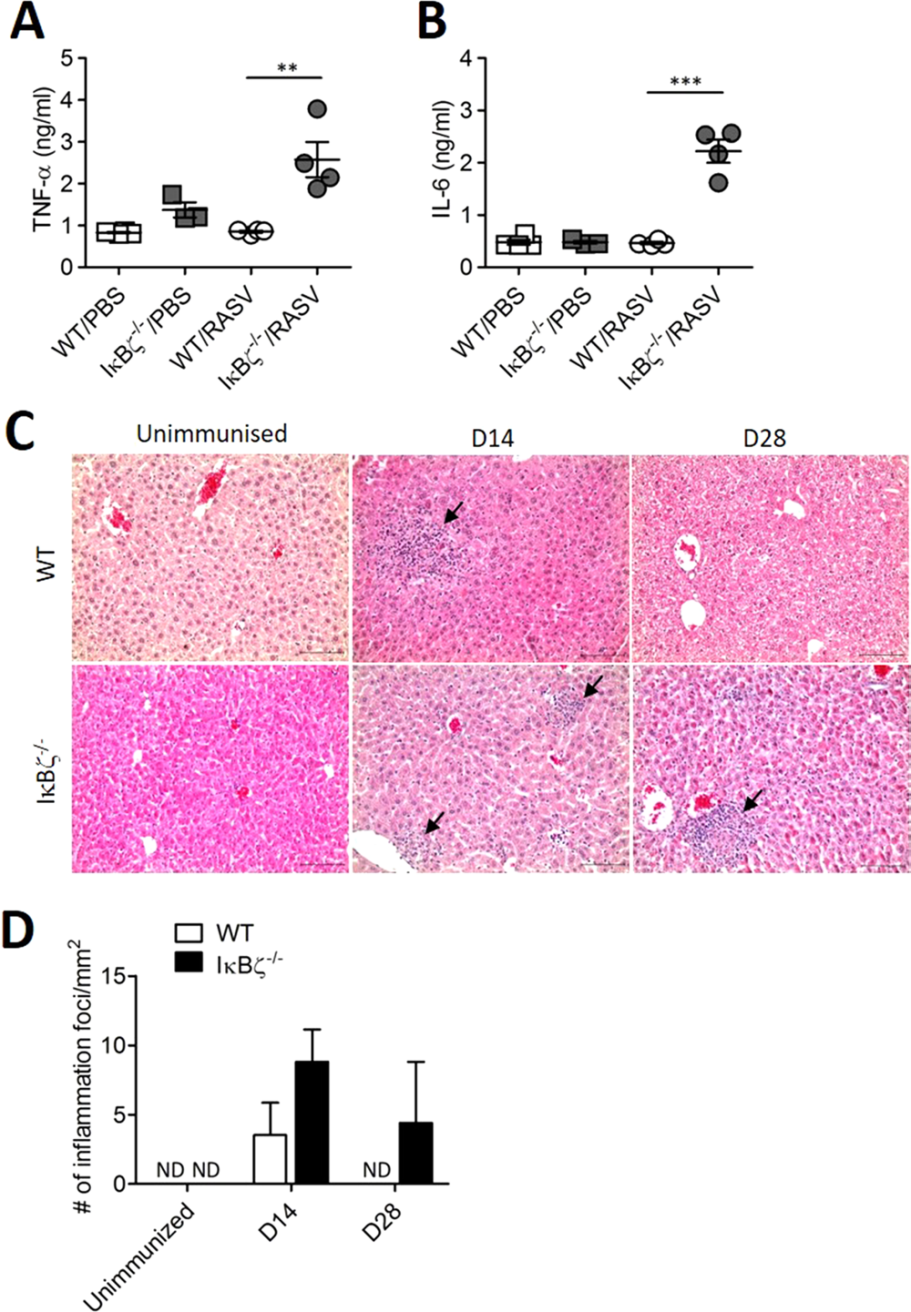
IκBζ^−/−^ mice exhibited enhanced inflammation after oral administration of a RASV strain. Wild-type and IκBζ^−/−^ mice were immunised with oral administration of 10^9^ CFU of RASV per mouse twice at 2-week intervals. (**A**,**B**) Inflammatory cytokines in the sera were analysed 14 d after the second immunisation (D28). Levels of TNF-α and IL-6 in the serum obtained from WT/PBS, IκBζ^−/−^/PBS, WT/RASV and IκBζ^−/−^/RASV were measured by cytokine bead array (CBA). ^**^P < 0.01 and ^***^P < 0.001 based on ANOVA with Bonferroni’s multiple comparison test. (**C**) Histopathology of the livers of wild type (WT) and IκBζ knock-out (IκBζ^−/−^) mouse on day 14 and day 28. Note the mononuclear inflammatory cell foci (arrows) in the liver parenchyma which were evident in the livers of both types on day 14 but only in the IκBζ^−/−^ mouse liver on day 28. H&E. Bars mean 100 μm. (**D**) The number of immune cell infiltration lesions was counted from histological liver examination images. Data are representative of three independent experiments. ND, not detected.

Because it was previously reported that IκBζ^−/−^ mice have highly elevated levels of cytokines, including IL-6 and IL-17^17,18^, the levels of inflammatory cytokines in the serum of IκBζ^−/−^ mice were determined after RASV administration. The levels of TNF-α, and IL-6 were significantly increased in IκBζ^−/−^ mice after oral administration with RASV (Fig. 2A,B). We assumed that high level of inflammatory cytokine (TNF-α and IL-6) of IκBζ^−/−^ mice could be associated with higher RASV burden due to insufficient induction of RASV-induced immune responses.

To compare the severity of inflammation after oral administration of the RASV strain, liver tissues from wild-type and IκBζ^−/−^ mice were collected at unimmunised and 14 d post-administration with RASV (D14) and 14 d after the second administration (D28). The histology of liver tissues showed inflammatory foci comprising infiltrated immune cells following administration of RASV (Fig. 2C). The inflammatory foci could be found in both wild-type and IκBζ^−/−^ mice at D14 but only in IκBζ^−/−^ mice at D28 (Fig. 2D). Taken together, these results suggested that IκBζ^−/−^ mice could not efficiently eliminate attenuated *Salmonella* after oral infection, although IκBζ^−/−^ mice exhibited increased inflammation following RASV administration.

### Oral immunisation with RASV failed to elicit an LPS-specific IgG response in IκBζ^−/−^ mice

**Figure 3:**
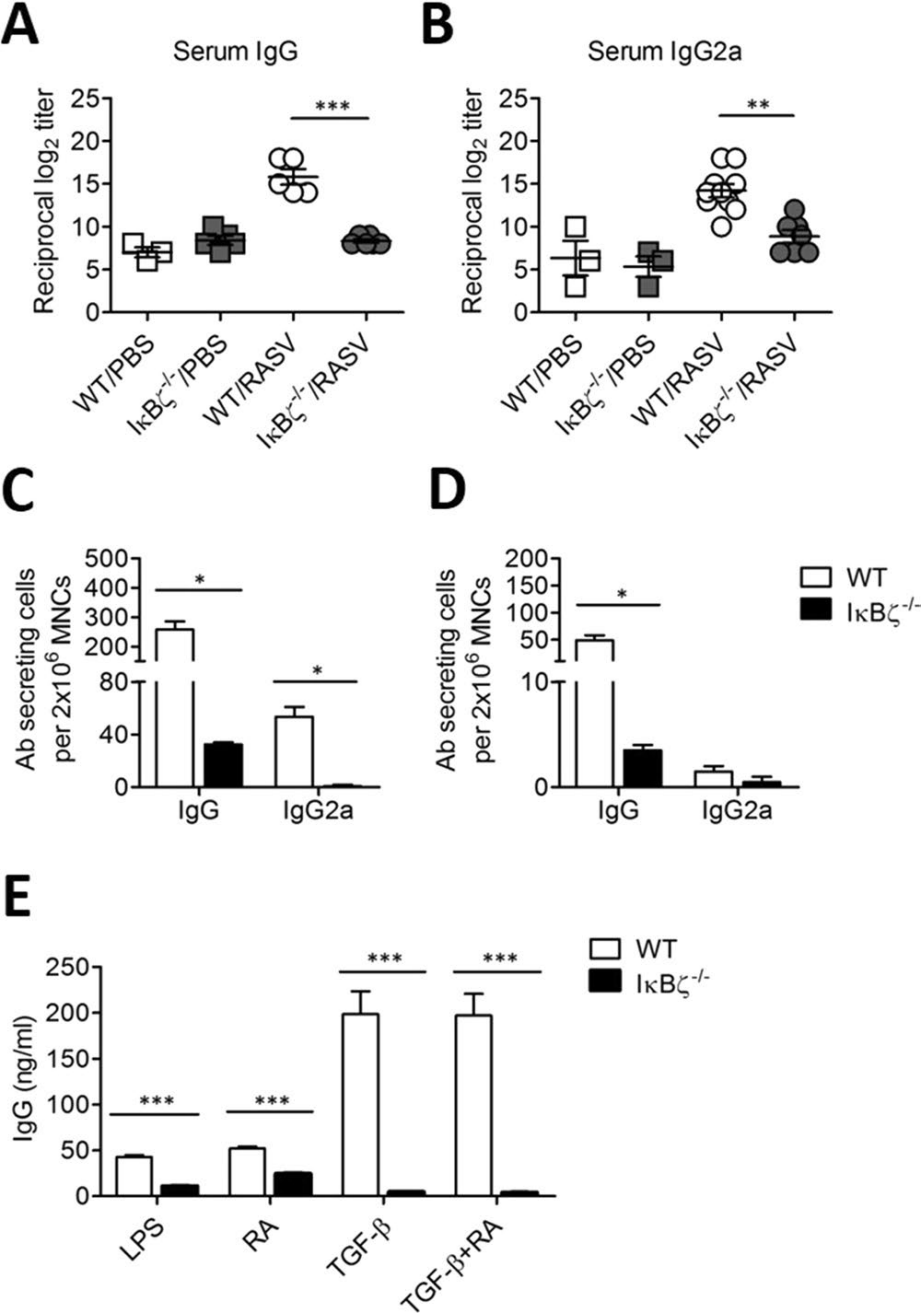
*Salmonella* LPS-specific IgG responses were significantly decreased in IκBζ^−/−^ mice following oral administration of a RASV strain. Wild-type and IκBζ^−/−^ mice were immunised orally with 10^9^ CFU of a RASV strain per mouse twice at 2-week intervals (n > 3 per group). (**A**,**B**) The level of *Salmonella* LPS-specific Ab in serum was measured at 14 d after the second immunisation. To titrate the levels of LPS-specific IgG and IgG2a, LPS-specific IgG and IgG2a antibodies were measured from the serum of WT/PBS, IκBζ^−/−^/PBS, WT/RASV and IκBζ^−/−^/RASV mice 14 d after the second immunisation. ^**^P < 0.01 and ^***^P < 0.001 based on ANOVA with Bonferroni’s multiple comparison test. (**C**,**D**) The number of LPS-specific Ab-secreting cells isolated from spleen (**C**) and mesenteric lymph node (**D**) was measured by ELISPOT assay 14 d after the second immunisation with the RASV strain. (**E**) Splenic resting B cells were cultured in the presence of 12.5 µg/ml LPS plus 25 nM of retinoic acid (RA) and/or 0.2 ng/ml of TGF-β for 7 d to induce Ab class switching. Total IgG levels were measured by ELISA in the culture supernatant. Data are representative of three independent experiments. ^**^P < 0.01 and ^***^P < 0.001 based on unpaired t-test.

A previous study reported that IκBζ plays a vital role in B cells, especially the LPS-mediated T cell-independent B cell class switching response (CSR)^19^. Thus, we analysed the levels of antigen-specific Ab production in IκBζ^−/−^ mice after oral RASV administration. *Salmonella* LPS-specific IgG levels, especially the IgG2a level, were significantly lower in IκBζ^−/−^ mice than in wild-type mice following RASV immunisation (Fig. 3A,B). Consistent with this, the numbers of *Salmonella* LPS-specific IgG- or IgG2a-secreting cell significantly decreased in the spleen and MLN (Fig. 3C,D).

To determine whether IκBζ^−/−^ B cells have an intrinsic defect regarding the induction of IgG production, resting B cells from wild-type and IκBζ-deficient spleens were stimulated with LPS in the presence or absence of retinoic acid (RA) and/or TGF-β to induce IgG class switching. IκBζ^−/−^ B cells failed to induce IgG production in all cases (Fig. 3E). These results suggested that IκBζ^−/−^ B cells had an intrinsic defect in the induction of Ab class switching. Therefore, the decreased production of LPS-specific IgG Ab in IκBζ^−/−^ mice following RASV immunisation could not control an infectious pathogen or even an attenuated vaccine strain.

### Germinal centre reaction after oral administration of RASV strain in IκBζ^−/−^ mice

**Figure 4:**
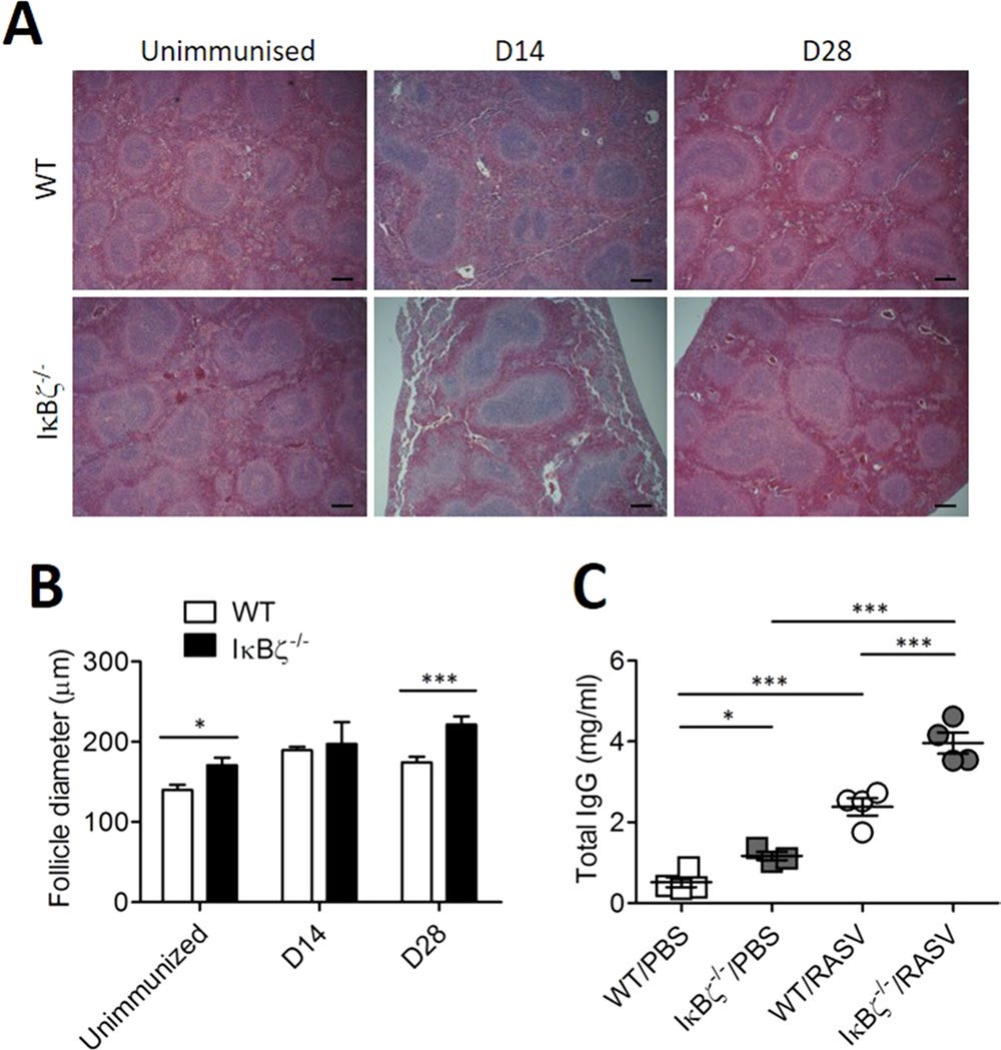
Germinal centre reaction after oral administration of a RASV strain *in vivo*. Wild-type and IκBζ^−/−^ mice were orally immunised with 10^9^ CFU of a RASV strain per mouse twice at 2-week intervals. (**A**) Representative H&E image of the spleen from an unimmunized mouse, 14 d after the first RASV oral administration (D14) and 14 d after the second RASV oral administration (D28). (**B**) Splenic follicle size was measured from histologic images. (**C**) Fourteen days after the second oral immunisation with RASV, the total IgG Ab level was measured in the serum. Data are representative of three independent experiments. ns, not significant; ^*^P < 0.05 and ^***^P < 0.001 based on ANOVA with Bonferroni’s multiple comparison test.

In our previous study, oral administration of RASV in Myd88-deficient mice resulted in chronic infection accompanied by enlarged germinal centres and hypergammaglobulinemia^15^, with increased levels of LPS-specific IgG responses^14^. Because Myd88 plays a crucial role in the stabilisation of IκB-ζ mRNA^20^, we checked the germinal centres and total IgG levels in IκBζ^−/−^ mice after RASV administration. Although IκBζ^−/−^ mice could not produce sufficient LPS-specific IgG Ab (Fig. 3A–D), the size of the germinal centres found in the spleen increased in IκBζ-deficient mice compared with those in wild-type mice after RASV administration (Fig. 4A,B). Moreover, the levels of total IgG also highly increased in the serum of IκBζ-deficient mice after RASV administration (Fig. 4C). Taken together, IκBζ^−/−^ mice failed to produce protective Ag-specific Ab and were finally infected even by an attenuated bacterial strain despite exhibiting a high degree of inflammation.

### IFN-γ producing Th1 responses were impaired in IκBζ^−/−^ mice

**Figure 5:**
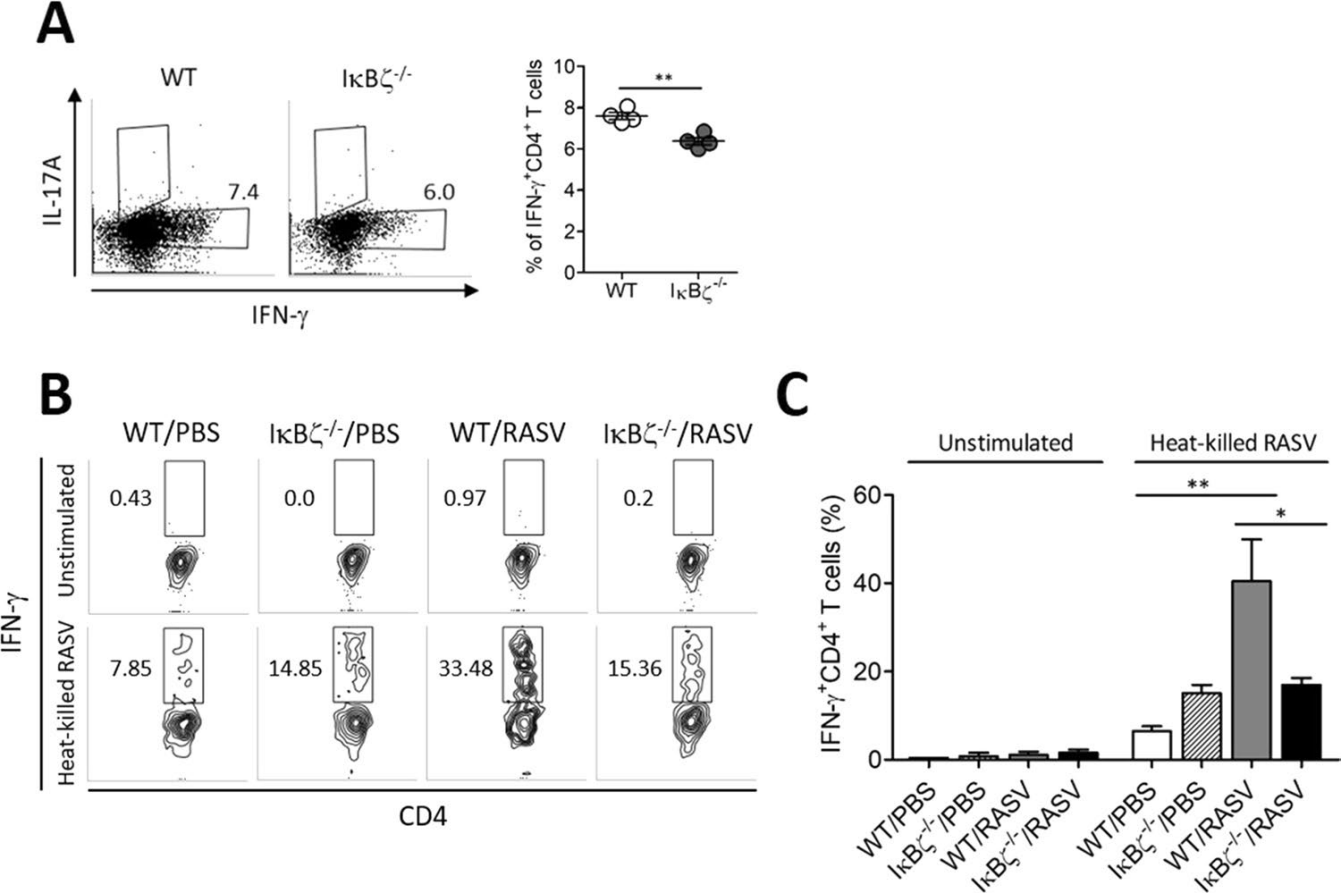
IFN-γ producing Th1 responses were impaired in IκBζ^−/−^ mice. (**A**) Naïve CD4^+^ T cells were cultured for 5 d with 2 µg/ml anti-CD3 and anti-CD28 antibody as induced to differentiate into Th1 cell with 10 ng/ml of IL-12. Under Th1 differentiation conditions, the intracellular IFN-γ on CD4^+^ T cells were analysed. ^*^P < 0.05 and ^**^P < 0.01 based on unpaired t-test. (**B**,**C**) Splenocytes isolated from RASV-immunised wild-type and IκBζ^−/−^ mice were re-stimulated by co-culturing with 10 heat-killed RASV per cell for 3 d. IFN-γ produced by CD4^+^ T cells were measured. (**B**) Representative intracellular staining results of IFN-γ synthesised by CD4^+^ T cells. (**C**) The percentages of IFN-γ-producing CD4^+^ T cells are shown. Data are representative of three independent experiments. ^*^P < 0.05 and ^**^P < 0.01 based on ANOVA with Bonferroni’s multiple comparison test.

According to a recent study, IκBζ^−/−^ T cells cannot differentiate to the Th17 subset because the interaction between the transcription factor RORγ complex and IκBζ is needed for differentiation to Th17 cells^3^. When we analysed T cells and B cells, the percentages of T cells and B cells in IκBζ^−/−^ mice are not significantly different with that of WT mice (Supplementary Fig. 2). To determine whether IκBζ^−/−^ CD4^+^ T cells have some defect in differentiating into the helper T cell lineage, naïve CD4^+^ T cells were cultured *in vitro* with conditioned media for each helper T cell lineage. IκBζ^−/−^ CD4^+^ T cells exhibited decreases in both IFN-γ-producing Th1 cells compared to the levels in wild-type cells (Fig. 5A,B). The Th1 response, as well as *Salmonella* LPS-specific Ab production, have been revealed to be essential for successful protection against *Salmonella* infection^21^. IκBζ^−/−^ mice produced markedly decreased levels of LPS-specific IgG2a Ab following RASV immunisation (Fig. 3C). To determine whether the reduced IgG2a production could be due to a reduced Th1 response, the levels of IFN-γ secreting CD4^+^ T cells were analysed following heat-killed RASV stimulation of splenocytes isolated from RASV immunised mice. Wild-type CD4^+^ T cells successfully induced IFN-γ production following heat-killed RASV re-stimulation (Fig. 5B,C). However, the IFN-γ-producing CD4^+^ T cells were significantly decreased in the IκBζ^−/−^ mice (Fig. 5B,C). These results suggested that IκBζ is crucial for the Ag-specific Th1 cell response and further production of Ag-specific IgG, especially IgG2a Ab. IκBζ deficiency failed to resist *Salmonella* infection even with an attenuated strain due to a decreased Th1 response followed by decreased levels of IgG2a.

## Discussion

IκBζ^−/−^ mice exhibit severe and chronic head and cervical inflammation, especially in aged mice^17,22^. Thus, they are widely used as an animal model of Sjögren syndrome^22^. One of the most common symptoms of Sjögren syndrome is mucosal dryness, which disrupts the mucosal barrier function. Because of this impaired host barrier function, mice with IκBζ deficiency may be more susceptible to infection by pathogenic organisms^18^. We hypothesised that the persistent invasion of exogenous microorganisms could be a trigger for the chronic inflammation. Furthermore, failure in innate and adaptive immunity could contribute to the chronic inflammation in IκBζ^−/−^ mice.

IκBζ plays a critical role in eradicating pathogens such as *Streptococcus pneumoniae* and *Legionella pneumophila*^23^. Individuals with a single nucleotide polymorphism in the IκBζ allele are more likely to succumb to pneumococcal infection^6^, and IκBζ regulates the expression of IL-6 in human monocytes in response to D39, a wild-type strain of *Streptococcus pneumoniae*^23^. In the current study, we found that mice with IκBζ deficiency developed chronic inflammation but failed to mount specific immune responses, including LPS-specific Ab production and *Salmonella*-specific T cell responses.

In addition to adaptive immunity induced by the RASV vaccination, innate immunity associated with IκBζ could affect on the protection against *Salmonella*. To confirm this, we performed *Salmonella* infection in bone marrow derived macrophages obtained from WT and IκBζ^−/−^ mice, and found that higher bacterial colonies were detected in IκBζ^−/−^ mice as compared to WT mice (Supplementary Fig. 7). This means that macrophage can play a role in *Salmonella* suppression in an IκBζ-dependent manner at early stage of infection.

To clarify the role of IκBζ in the induction of acquired immunity, we used a live attenuated recombinant *Salmonella* vaccine strain (RASV) to induce *Salmonella*-specific T cell and B cell responses. Typically, *Salmonella* invades via the gastro/oral route through contaminated food or water, causing salmonellosis accompanied by fever and diarrhoea^14,15^. *Salmonella* organisms possess a variety of antigenic molecules such as LPS, flagellin, and lipoprotein, which can also bind to TLR to trigger the innate immune responses^24–26^. Previously, we showed that Myd88 deficiency in mice mediated hypergammaglobulinemia with increased LPS-specific IgG Ab after oral administration of RASV^15^. Furthermore, we showed that WT mice which were adoptively transferred with sera from RASV-vaccinated Myd88^−/−^ mice were partly protected against pathogenic *Salmonella* infection^14^. Contrary to Myd88, it was suggested that IκBζ is a critical regulator of TLR-mediated class switch recombination (CSR) in B cells, and mice with IκBζ deficiency have impaired type 1 T cell-independent Ab responses^19^; thus, we presumed that the LPS-specific response might be reduced after RASV administration in IκBζ^−/−^ mice. Indeed, the levels of LPS-specific Ab responses in RASV-vaccinated IκBζ^−/−^ mice were significantly lower than those in RASV immunised WT mice. However, the levels of total IgG were markedly increased in RASV-vaccinated IκBζ^−/−^ mice compared to those in RASV-vaccinated WT mice. Thus, we confirmed that the LPS-specific Ab response, which represents the type 1 T cells-independent Ab response, was impaired in IκBζ^−/−^ mice after RASV vaccination. To check how much *Salmonella*-specific IgG was in the total IgG of RASV-vaccinated IκBζ^−/−^ mice, we conducted ELISA using whole cells of RASV as an antigen. The levels of anti-RASV IgG were higher in the serum of RASV-vaccinated IκBζ^−/−^ compared with those in the serum of RASV-vaccinated WT mice (Supplementary Fig. 3). These data suggest that T cell-dependent and type 2 T cell-independent antibody production responses may occur in IκBζ deficiency after RASV vaccination. However, the higher levels of total antibody and RASV-specific antibody did not confer a protective effect against lethal *Salmonella* challenge.

The type 1 helper T cell (Th1) response is strongly induced in the host after *Salmonella* infection^21^. Th1 cells secrete interferon-γ, which activates macrophages and monocytes and makes them more potent in capturing and digesting *Salmonella*^21,27,28^. In the current study, we found that naïve CD4^+^ T cells from IκBζ^−/−^ mice differentiated less to Th1 cells under IL-12-dependent differentiation conditions than WT CD4^+^ T cells. Furthermore, the secretion of IFN-γ by CD4^+^ T cells from RASV-vaccinated IκBζ^−/−^ mice was lower than that by cells from RASV-vaccinated WT mice after restimulation with heat-killed RASV. In a previous study, it was shown that IκBζ regulated the production and secretion of IFNγ in human NK cells after stimulation with IL-12 and IL-18^27,29^. Likewise, it was suggested that the production of IFN-γ was decreased by IκBζ deficiency in a macrophage cell line after stimulation with IL-1β and IL-18^30^. These findings showed that IκBζ binds to the NF-κB binding site of the IFN-γ promoter to switch on IFN-γ mRNA transcription^29^. Collectively, the T cell-intrinsic activation of the IκBζ molecule after stimulation with IL-1 may be necessary for the differentiation of naïve CD4^+^ T cells into IFN-γ-secreting Th1 cells and play an essential role as a transcription factor to trigger IFN-γ production.

In addition, IκBζ mediates mucosal barrier function through secretion of inflammatory cytokines in epithelial cells^7,22,23,31,32^. Thus, it was supposed that the weaker barrier function of IκBζ^−/−^ mice allowed the invasion of commensal bacteria, which might also influence the occurrence of chronic inflammation. In our study, we administered RASV via the oral route, and disseminated RASV via a leaky gut might induce systemic infection. In addition, we found that higher levels of IFN-γ-positive cells are found in non-vaccinated IκBζ^−/−^ mice as compared to those in non-vaccinated WT mice. We presume that the impaired mucosal barrier function of IκBζ^−/−^ mice could lead higher level of IFN-γ producing CD4^+^ T cell against some commensal bacteria which genetically close with *Salmonella* organism.

Nramp1 is a well-known factor for Fe^2+^-dependent reactive oxygen species production by phagocytes, and the 129 Sv mice having Nramp1^G169^ are more resistant to *Salmonella* than C57BL/6 mice having homozygous mutation of Nramp1^G169D^ allele^33–36^. Thus, in the current study, we confirmed that both of mixed-background 129Sv-ICR IκBζ^−/−^ mice and littermate WT control mice have susceptible Nramp1^G169D^ allele (Supplementary Fig. 8).

In the current study, we hypothesised that IκBζ^−/−^ mice could be protected from pathogenic *Salmonella* infection through immunisation. However, the oral administration of RASV to IκBζ^−/−^ mice did not result in the production of high levels of LPS-specific antibody, and the mice were more susceptible to challenge with a lethal strain of *Salmonella* because of impaired T cell and B cell responses. Therefore, we presume that the reduced expression or the defective function of IκBζ in some populations might be associated with their higher susceptibility to specific pathogenic infections and that they can also be infected by the administration of an attenuated strain of bacteria which is given for vaccination. Thus, the administration of live attenuated vaccine strains should be avoided or re-considered for individuals with insufficient induction of adaptive immune responses due to an IκBζ hypomorphic mutation. Overall, we suggest that IκBζ is critical to mediate vaccine-induced adaptive immunity as well as innate immunity.

## Materials and Methods

### Mice and bacteria

IκBζ^−/−^ mice were provided by Prof. Shizuo Akira (IFReC, Osaka University, Japan)^18^. It was known that inbred C57BL/6 genetic background IκBζ^−/−^ mice is embryonic lethal^3^. Thus we used IκBζ^−/−^ mice on a mixed 129Sv-ICR mice. All experiments were conducted with IκBζ^−/−^ and their littermate WT control mice obtained from mating between mixed 129Sv-ICR IκBζ^+/−^ mice. The mice used in this study were maintained in an experimental facility of Kangwon National University under specific pathogen-free conditions. All animal experiments, including the RASV immunisation and UK-1 challenge experiment, were approved by the Institutional Animal Care and Use Committees (IACUC) of Kangwon National University (Permit Number: KW-160201-2) and were performed in accordance with approved guidelines and regulations. Attenuated *Salmonella* *typhimurium* χ9241 (*ΔpabA1516 ΔpabB232 ΔasdA16 ΔaraBAD23 ΔrelA198:: ara*CP_BAD_*lacI* (ATG)TT containing pYA3620) and the virulent *Salmonella* strain UK-1 used in this study were kindly provided by Roy Curtiss, III (Arizona State University). All strain of bacteria was cultured in Luria-Bertani media at 37 °C in a shaking incubator, and prepared as previously reported^14^. For immunisation, RASV (10^9^ CFU/mouse) was orally administered and secondly immunised at the same dose after 14 d as the second immunisation. UK-1 was challenged at a dose of 10^7^ CFU/mouse 14 d after the final RASV oral administration. We confirmed that all mice were died by administration of 2 × 10^6^, 10^7^, and 5 × 10^7^ CFU per mouse (Supplementary Fig. 4). The administration of *Salmonella* doses was checked by plating serial dilutions onto XLD agar (Becton, Dickinson, MD, USA) plates.

### ELISA and ELISPOT

ELISA and ELISPOT were conducted according to a previous study^14,37^. Briefly, 5 μg/ml *Salmonella* *typhimurium*-derived LPS (Sigma-Aldrich) in 50 mM sodium bicarbonate (Sigma) was coated on 96-well immunoplates (Falcon) and incubated overnight at 4 °C. Next, the immunoplates were aspirated and washed 3–5 times using 1X PBS containing 0.05% Tween-20 (1X PBS-T). After the washing step, immunoplates were blocked with 1% BSA for 2 h at 37 °C, and samples were added to plates with samples diluted 2-fold serially starting with a 1/16 dilution in 0.1% BSA, followed by incubation for 2 h at 37 °C. Goat anti-mouse IgG, and IgG2a antibodies conjugated with HRP (Southern Biotechnology Associates) were diluted at a 1:5000 ratio in 1X PBS-T and added to each well, followed by incubation at 37 °C. For colour development, the substrate solution (TMB, Surmodics) was added, and the reaction was stopped by adding 0.5 N HCl. The colour development was measured at 450 nm on an ELISA reader (Microplate spectrophotometer; Molecular Devices), and antibody titres were calculated as reciprocal log_2_ titres.

For ELISPOT assays, 5 μg/ml *Salmonella* *typhimurium*-derived LPS (Sigma-Aldrich) in 50 mM sodium bicarbonate (Sigma) was coated on 96-well nitrocelluose immunoplates and incubated overnight at 4 °C. Next, the immunoplates blocked with RPMI 1640 (Gibco) supplemented with 10% foetal bovine serum (Gibco), and serially diluted mononuclear cells were plated. After incubation for 4 h at 37 °C in a 5% CO_2_ incubator, 1:5000 diluted goat anti-mouse IgG, and IgG2a antibodies conjugated with HRP (Southern Biotechnology Associates) were applied to each well. For colour development, a peroxidase substrate (3-amino-9-ethylcarbazole kit; Moss) was applied, and the number of Ab-secreting cells was counted with the aid of a stereomicroscope (SZ2-ILST; Olympus).

### *In vitro* CD4^+^ T cell differentiation

To prepare primary lymphocytes, spleen, mesenteric, inguinal, axillary, and superficial cervical lymph nodes were obtained from WT and IκBζ^−/−^ mice and mechanically ground through a nylon mash. The ground tissue was added to ACK lysing buffer to remove red blood cells. CD4^+^ T cells were negatively selected using a CD4^+^ T cell isolation kit (Miltenyi Biotech) and stained with a fluorescence-conjugated antibody. CD25^−^CD4^+^CD44^low^CD62L^hi^ naïve CD4^+^ T cells were sorted using an Aria II instrument (Becton, Dickinson, MD, USA) in the Central Laboratory of Kangwon National University. Gating strategy was shown in Supplementary Fig. 5. Then, 10^5^ naïve CD4^+^ T cells were seeded onto 96 well plates pre-coated with anti-CD3, and anti-CD28 antibody with RPMI 1640 media supplemented with 10% FBS (base condition media). Conditioned media contained 10 ng/ml IL-2, 10 ng/ml IL-12, and 10 µg/ml anti-IL-4 Ab for Th1. After 5 d of culture at 37 °C in a 5% CO_2_ incubator, cells were stimulated with 50 ng/ml PMA, 1 µg/ml ionomycin, and brefeldin A for 4 h. Cells were analysed with a FACSverse instrument and Flowjo program.

### Stimulation with heat-killed RASV

To inactivate RASV bacteria, bacteria cell suspension was incubated on 100 °C and 10 min. The spleens from unimmunised and RASV-immunised mice were harvested at 14 d after the second immunisation. Splenocytes were seeded at 2 × 10^5^ cells/well on 96 well cell culture plates and re-stimulated with 10 heat-killed bacteria per cell of RASV for 3 d at 37 °C in a 5% CO_2_ incubator. Before harvest, 5 μg/ml of brefeldin A was added into cell culture media. Harvested cells were stained with PerCP-cy5.5 conjugated anti-mouse CD4 antibody and LIVE/DEAD^TM^ Fixable Near-IR Dead cell staining kit separating live and dead cell (Invitrogen^TM^). After surface marker staining, intracellular cytokine staining was conducted with IC Fixation buffer and intra-cellular IFN-γ was stained with APC-conjugated anti-mouse IFN-γ antibody according to the manufacturer’s protocol (Invitrogen^TM^).

### *In vitro* class switching response

Splenic resting B cells were isolated using CD43 microbeads (Miltenyi Biotech) in accordance with the manufacturer’s procedure. A total of 5 × 10^5^ CD43^+^ B cells were seeded on a 24 well plate and cultured for 3 d with 12.5 µg/ml LPS or in a combination of 25 nM retinoic acid and/or 0.2 ng/ml TGF-β. Cultured supernatants were analysed for the concentration of polyclonal IgG Ab level by ELISA.

#### Statistical analysis

To compare the differences between the two experimental groups, we used the Student’s *t-*test. We compared multiple groups using a one-way analysis of variance (ANOVA) followed by Bonferroni’s multiple comparison test using GraphPad Prism version 5 (GraphPad Software, La Jolla, CA, USA). Values of *P* < 0.05 were considered significant at a 95% confidence interval. Survival (Kaplan-Meier) curves were compared using the log-rank test (GraphPad Prism version 5 (GraphPad Software, La Jolla, CA, USA)).

## Supplementary information

Dataset 1
