# Supplementary material for: IκBζ facilitates protective immunity against Salmonella infection via Th1 differentiation and IgG production
Source: Sci Rep. 2019 Jun 10;9:8397. doi: 10.1038/s41598-019-44019-3 (PMC6557891; doi:10.1038/s41598-019-44019-3)
Supplement: Supplementary file 1 — Dataset 1 [file 41598_2019_44019_MOESM1_ESM.pdf]

IκBζ facilitates protective immunity against *Salmonella* infection via Th1 differentiation and IgG production

Jae-Hee Ahn[1], Jaewon Cho[1], Bo-Eun Kwon[1], Geun-Shik Lee[2], Sung-il Yoon[3], Seung Goo Kang[3], Pyeung-Hyeun Kim[4], Mi-Na Kweon[5], Hyungjun Yang[6], Bruce A Vallance[6], Young-In Kim[7], Sun-Young Chang[7], Hyun-Jeong Ko[1]

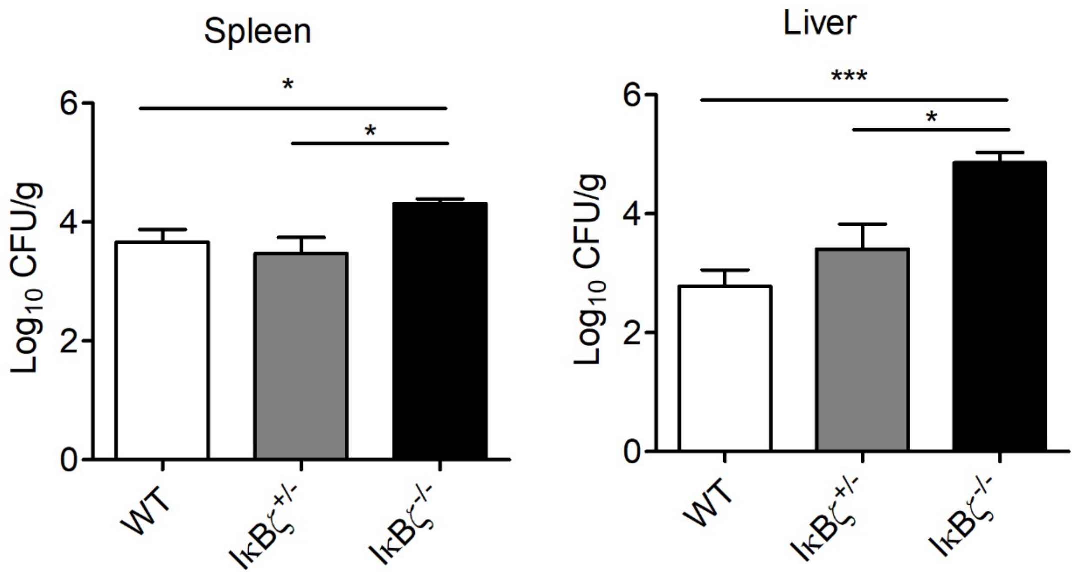

Supplementary Figure 1

Colony forming units of *Salmonella* UK-1 strain were counted with tissue homogenates obtained from liver and spleen at 9 days after oral infection of 10^7 CFU/mouse. Tissue homogenates was applied onto XLD agar plate and incubated about 24 hours. *P < 0.05 and ***P < 0.001 based on unpaired t-test.

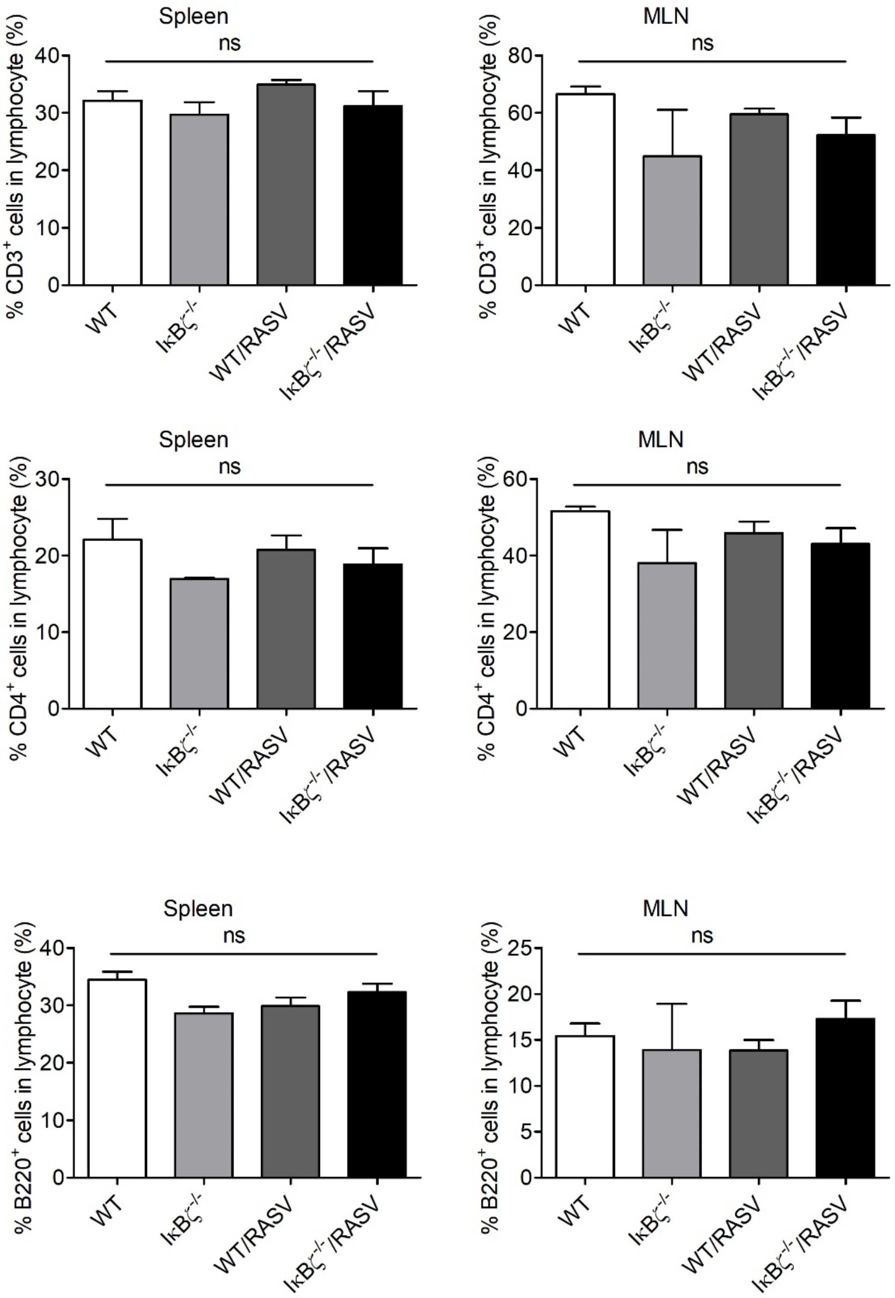

Supplementary Figure 2

$CD3^+$, $CD4^+$ and $B220^+$ cells from spleen and lymph nodes were analyzed through flow cytometry. Spleen and mesenteric lymph nodes (MLN) were obtained after two-times administration of RASV with 14 days interval. ns, not significant; based on ANOVA with Bonferroni's multiple comparison test.

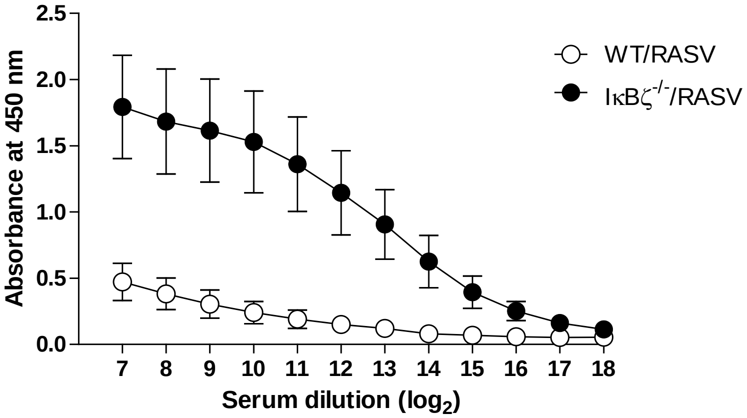

Supplementary Figure 3

Levels of *Salmonella*-specific IgG were evaluated by ELISA using whole cells of RASV as an antigen. Sera were obtained from groups of mice after two-times immunizations with RASV. For quantification of RASV-specific antibody in mouse serum, 10^7 CFU/well of RASV-whole cell bacteria were coated onto 96 well plate overnight. Further step was proceeded as described in method. Sera were next diluted by two-fold starting from 1:200 dilution.

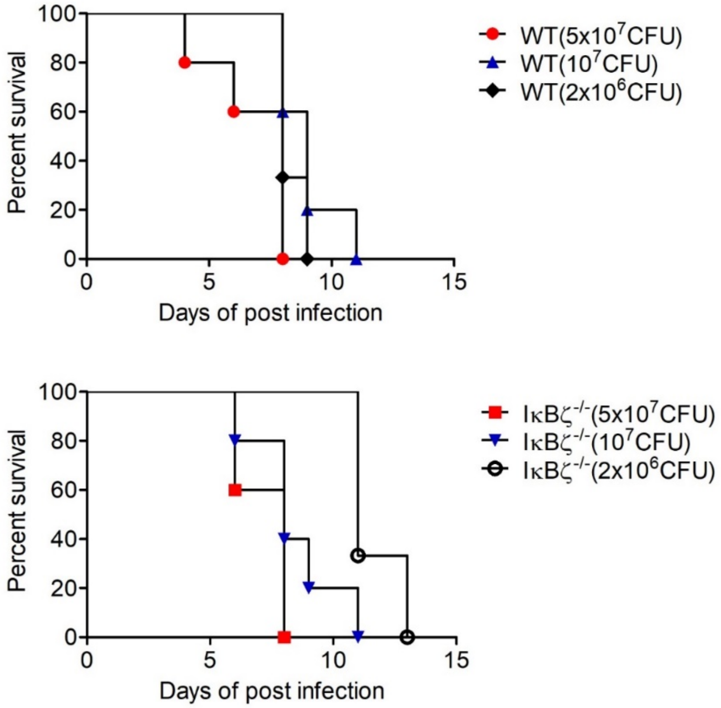

Supplementary Figure 4

Wild-type (WT) and IκB$\zeta^{-/-}$ mice were orally challenged with 2 x 10^6, 10^7, and 5 x 10^7 CFU of a lethal wild-type *Salmonella* strain (UK-1) per mouse.

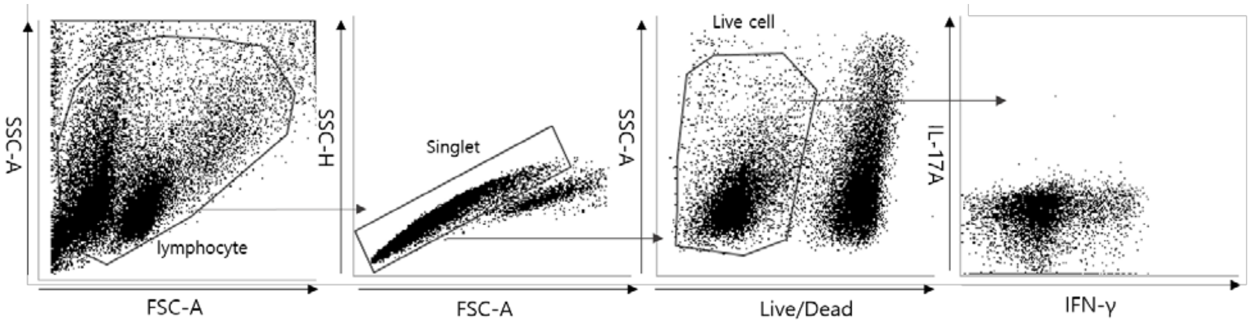

Supplementary Figure 5

Gating strategy to characterize interferon-γ and IL-17A producing cells differentiated by 10 ng/ml of IL-12.

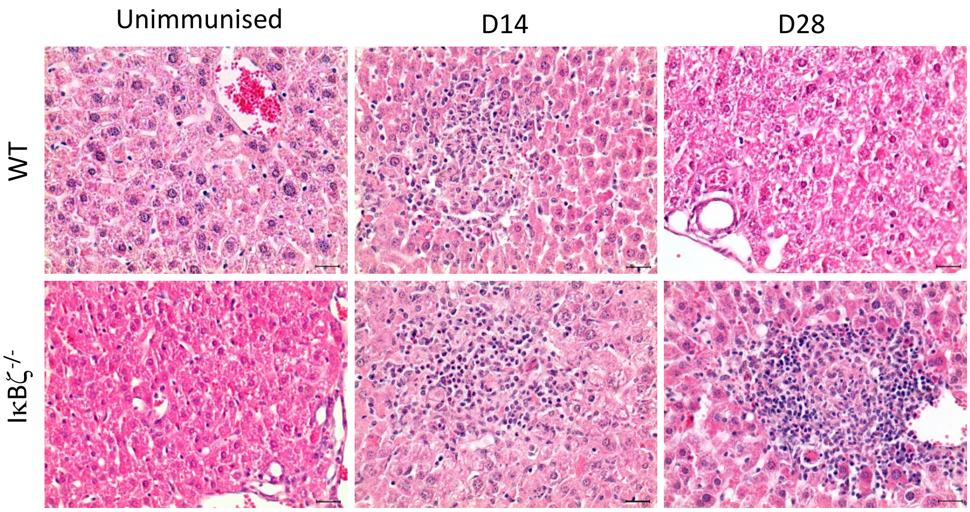

Supplementary Figure 6

High-magnified pictures of Figure 2C. Scale bar is 25μm.

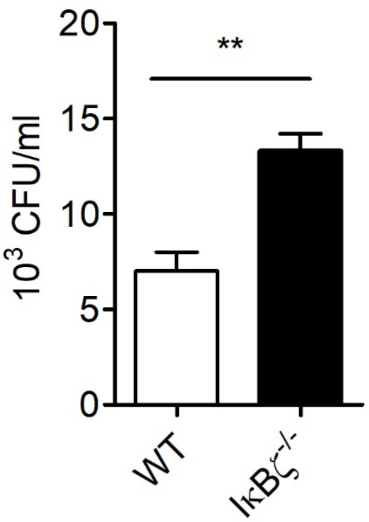

Supplementary Figure 7

Bone marrow derived macrophages (1 x 10^5 cells/ well) from WT and IκB$\zeta^{-/-}$ mice were co-cultured with 10 MOI of live UK-1 for 4 hours, and then lysed with PBS containing 1% Triton X-100. *Salmonella* CFU was checked by plating serial dilutions onto XLD agar plates.

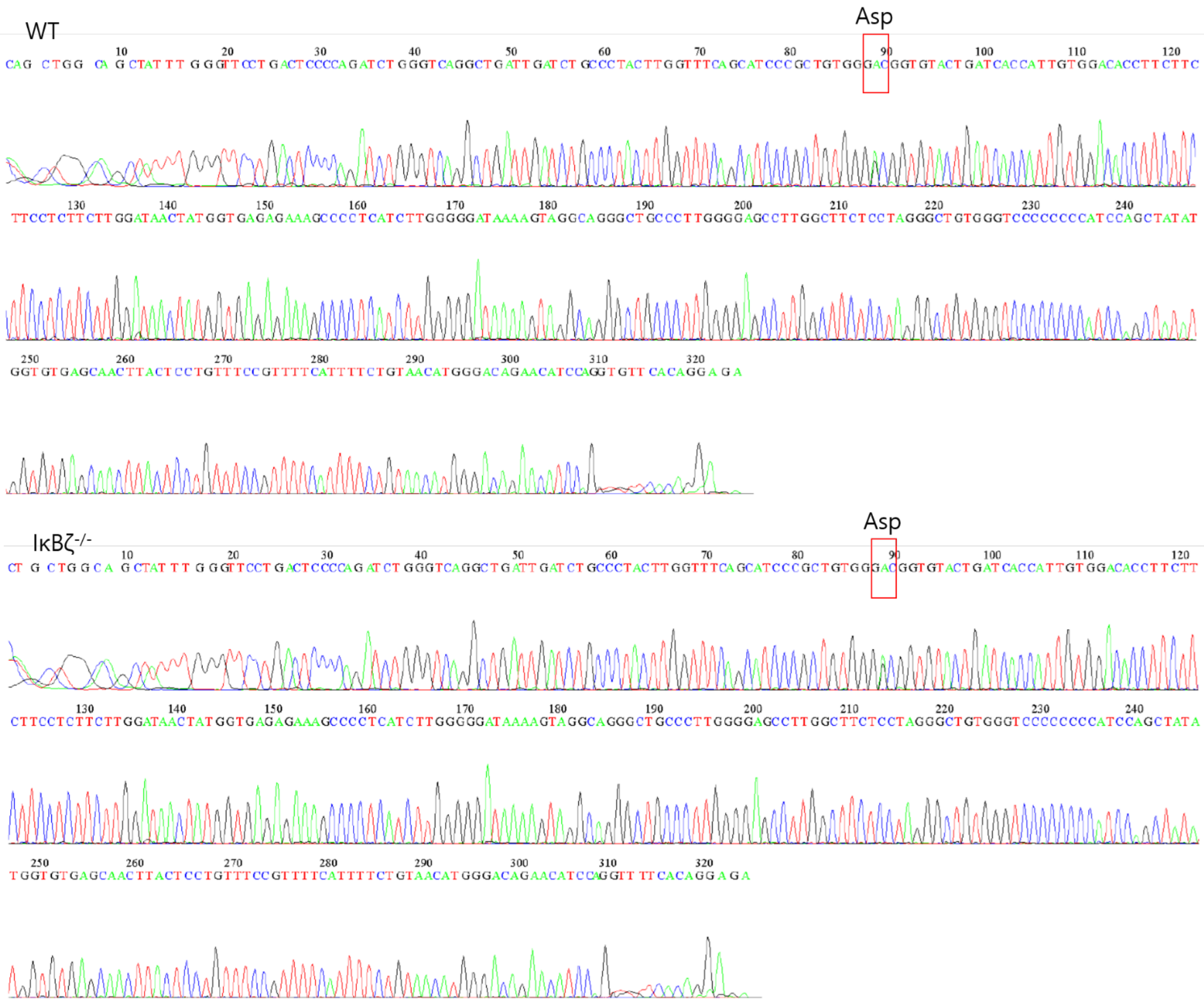

Supplementary Figure 8

The genetic status of Nramp1 was analyzed by genomic DNA sequencing. Tail DNA isolated from WT and IκBζ$^{-/-}$ mice were analyzed using the following primers; Forward 5’-TTC AAC ACA ACC CAC ACT CC-3’, Reverse 5’-CCT GTG ACA CCT GGA TGT TCT-3’.
